# Considerations for Carnosine Actions in Biology

**DOI:** 10.1007/s11064-026-04735-5

**Published:** 2026-03-27

**Authors:** Belisa Parmeggiani, Bruna Klippel Ferreira, Patricia Fernanda Schuck, Gustavo Costa Ferreira

**Affiliations:** Laboratório de Erros Inatos do Metabolismo, Instituto de Bioquímica Médica Leopoldo de Meis, Universidade Federal do Rio de Janeiro, Rio de Janeiro, Brazil

**Keywords:** Imidazole dipeptides, Stage of development, Biological variables, Therapeutic potential, Carnosinemia

## Abstract

Carnosine is a histidinic dipeptide mainly identified for its pH buffering, antioxidant and metal chelating capacities. Several studies have explored the potential benefits of carnosine as a supplement for exercise, as well as an adjuvant treatment in several pathologies; however, roles and impacts of carnosine on most tissues, including the brain, are still under debate, especially in earlier stages of development. There is evidence that carnosine may impact a myriad of physiological parameters. It includes potential roles of carnosine as a modulator of cell survival, redox homeostasis, signaling and metabolism, among other functions. Many variables seem to impact the outcomes of carnosine actions (e.g., carnosine concentrations, length of exposure, target cell/tissue, biological sex, metabolic state, and developmental stage). Considering that the physiology and metabolism of histidine dipeptides change throughout life, impacts of carnosine during development should be carefully considered. This is particularly relevant in light of carnosinemia, an inherited disorder of carnosine catabolism characterized by the accumulation of carnosine and presenting neuropsychomotor dysfunction. Thus, rethinking the applications of carnosine is crucial for realization of the full potential of this promising molecule.

## Introduction

Histidine dipeptides or imidazolic dipeptides are small molecules formed by L-histidine (or its methylated forms) and other amino acids [1]. Carnosine is an imidazolic dipeptide constituted by β-alanine and L-histidine. It was first described by Vladimir Gulevich in 1900 [2]. Other common histidinic dipeptides include homocarnosine (γ-aminobutyryl-L-histidine) and anserine (β-alanyl-N-π-methyl-L-histidine). These molecules (particularly carnosine) have been explored by the scientific community for their potential in the treatment of human diseases. However, carnosine actions on cellular homeostasis are still an understudied area. In this narrative review, we aim to explore some of the potential biological variables that may impact carnosine effects, especially at earlier stages of development.

## Carnosine: Distribution and Metabolism

Carnosine is widely distributed in rodents and humans, being detected at varying concentrations in muscles, brain, kidneys, heart, adipose tissue, liver, and lungs [3, 4]. In humans, carnosine is the most abundant histidine dipeptide in muscle [5]. Homocarnosine and anserine are also found at varying concentrations in different mammalian tissues [3, 6, 7].

Carnosine (or its constituent amino acids) may be taken up from the diet, mainly from beef, poultry, or fish [4, 5, 8]. Carnosine may also be endogenously synthesized by carnosine synthetase (EC 6.3.2.11), which is encoded by the *CARNS1* gene. Carnosine synthetase is found in the cytosol of cells from several tissues, including brain, muscle, kidneys, and liver [9]. Protein levels of carnosine synthetase correlate to some extent with tissue content of histidinic dipeptides [3, 10, 11]. On the other hand, serum carnosinase (CN1; EC 3.4.13.20) and cytosolic non-specific dipeptidase (CN2; EC 3.4.13.18) are the enzymes that catalyze the breakdown of carnosine into its constituent amino acids [12]. Unlike other histidinic dipeptides, carnosine hydrolysis mediated by CN2 is favored at non-physiological pH. Thus, CN1 activity with carnosine is considerably higher than CN2 activity [13–15]. CN1 expression is encoded by the *CNDP1* gene. In humans, CN1 is expressed in brain and liver (and mostly secreted to the cerebrospinal fluid and serum), whereas CN2 is expressed ubiquitously [15–17]. In rodents, CN1 expression is restricted to the kidneys [15], and carnosinase activity in other tissues is attributed to CN2 [18, 19]. Carnosine uptake can be mediated by different transporters. Peptide transporter 2 (PEPT2), which is encoded by the *PEPT2* gene, is expressed in several mammalian tissues, including kidneys [20, 21], skin [22], lungs [23], and heart [24]. In the brain, PEPT2 is found in cells of the choroid plexus [25], astrocytes [26, 27], and neurons [28]. Peptide transporter 1 (PEPT1), which is encoded by the *SLC15A1* gene, is the main transporter for carnosine in the small intestine [29–32]. The peptide/histidine transporters (PHT1, encoded by *SLC15A4*, and PHT2, encoded by *SLC15A3*) have also been demonstrated to take up carnosine [33] and are expressed in mammalian muscles [19, 34] and gut [32], as well as in glioblastoma [33]. Interestingly, PHTs (especially PHT1) may represent the main carnosine transporter in muscle, as this tissue shows negligible mRNA expression of PEPT1/2 [19].

## Physiological Roles of Carnosine

Most studies identify pH buffering as the main physiological role for carnosine. This is supported by the pKa for protonation of its imidazolic ring (pKa = 6.72) [35], as well as by the correlation between muscle carnosine content and tissue buffering capacity [3, 36, 37].

Carnosine has also been postulated to serve as an effective antioxidant. Carnosine interacts with reactive oxygen species and carbonyl groups, and provides protection against lipid oxidation in a variety of conditions (for a comprehensive review, see [38]). The antioxidant actions of carnosine can also be attributed in part to its role as metal ion chelator [39, 40], since bivalent metal cations (such as Cu^2+^, Zn^2+^ and Fe^2+^) may lead to the formation of reactive oxygen species through a variety of mechanisms [41, 42]. Calcium (Ca^2+^) handling in skeletal [43] and cardiac [44] muscles has also been attributed to carnosine.

## Impacts of Carnosine Supplementation on Cell Physiology

In addition to its physiological roles, there are many other effects that can be attributed to carnosine, including browning of adipocytes [45], induction of reactive morphology in astrocytes [46], and differentiation of C2C12 myoblasts [47]. Interestingly, carnosine exposure can lead to variable outcomes in the tested parameters. For example, carnosine can act as a pro-oxidant [46, 48–50] despite its widely reported antioxidant effect [51–53]. Similar variability is also observed for cell viability [52–61], cell proliferation [34, 62–65], metabolism [66–72], and other parameters [56, 73].

There are a number of factors that can modify the effects elicited by carnosine. For instance, alterations in redox and metabolic environment, as well as aging, can trigger an apparent duality in the effects of this dipeptide. In this scenario, the presence of hydrogen peroxide in the incubation medium alters carnosine effects from a pro-oxidant to an antioxidant profile in cultured astrocytes [46]. This is also seen with myoblasts: addition of carnosine to a homeostatic medium decreases cell viability, whereas carnosine improves cell viability in myoblasts exposed to hydrogen peroxide [74]. Impacts of carnosine on astrocytic mitochondrial function also differ whether glucose/oxygen deprivation conditions are present or absent [75]. A similar duality has also been observed in vivo: while carnosine has no impact on redox parameters in serum or liver of adult rats, carnosine decreases lipid peroxidation markers and increases antioxidant levels in older animals [76, 77]. These data suggest that biological variables such as redox status, metabolic status and age can modulate the effects of carnosine. In this context, it may be that other biological variables can also impact carnosine outcomes.

## Potential for Treatment of Human Diseases

The therapeutic potential of carnosine has been a topic of interest for some time [78, 79]. Clinical and pre-clinical studies have explored the possible use of carnosine (and its precursor β-alanine) as a treatment/adjuvant for different human diseases, including diabetes [80–83], cardiovascular disorders [81, 84], obesity [45, 81, 85], cancer [86, 87], COVID19 [88], psychiatric disorders [89–91], and Alzheimer’s and other aging-related diseases [80, 92, 93]. For instance, a double-blinded randomized control trial tested carnosine as a treatment for type 2 diabetes. Carnosine was given twice daily (1 g per administration; 2 g per day for 14 weeks) to 24 individuals (placebo group had 25 individuals). Pre-diabetic or diabetic adults who received oral carnosine supplementation exhibited improved glucose tolerance [94] (but cardiovascular and cardiometabolic risk factors in these individuals were not improved by carnosine [83]). Other studies testing similar carnosine administration regimens (1 g carnosine twice daily; 2 g per day for 12 weeks) showed that carnosine does not affect most of the plasma/serum and urinary biochemical indicators of kidney function and metabolic homeostasis, but it may improve selected parameters (e.g. by decreasing urinary TGF-β levels [82]), and in overweight patients it improves insulin sensitivity [95]). For a comprehensive review on the available clinical studies using carnosine, please refer to Saadati et al. [96] and Sureshkumar et al. [97]. Interestingly, a recent single-center, open-label dose escalation study indicated that carnosine appears to be safe and well tolerated up to a single dose of 10 g. However, at higher doses (15 g carnosine), participants experienced side effects (77% of 16 participants), including headache (43.5%), nausea (21.7%), and paresthesia (21.7%) [98].

## Muscle Carnosine and Exercise Performance

Several studies evaluate whether carnosine or β-alanine supplementation affect exercise performance. Carnosine has been proposed to improve exercise performance due to its classical pH buffering and antioxidant capacities. Balancing muscle pH and redox status in muscle is important to maintain tissue homeostasis (e.g., contractile capacity, tissue integrity, cell signaling, and others) [99, 100]. During exercise, pH and reactive species production may increase in blood and muscle due to the increased energy demand for muscle contraction [101]. Supplementation with carnosine (4 g/day for 14 days) prevents the increase of the levels of oxidative damage markers (8-isoprostane, 3-nitrotyrosine, oxidized glutathione) in plasma of professional male kayakers after exercise [102].

Supplementation with β-alanine, a precursor of muscle carnosine, has also been proposed. The rationale is based on the high CN1 activity found in humans, which favors a rapid degradation of most carnosine acquired in the diet. Indeed, β-alanine supplementation (in daily doses of 4.8 g to 6.4 g for 6 weeks) can increase muscle carnosine content in healthy adults [103, 104]. Supplementation with β-alanine is associated with better pH buffering capacity in blood, increased blood lactate, and delayed time to exhaustion during a cycling capacity test [104]. Improvement of redox homeostasis after exercise has also been reported in randomized, placebo-controlled trials among sedentary individuals receiving β-alanine [105]. However, other studies did not report beneficial actions of β-alanine in exercise performance [106–112].

## Carnosine and Biological Sex

There is evidence that carnosine metabolism and distribution may differ between sexes. Carnosine content is lower in kidney [113] and heart [114] of female mice. In mice, expression of *Carns1* and *Pept2* in kidney is higher in females than in males, while *Cndp1* expression is higher in males [115]. Aging decreases renal carnosine and anserine levels only in female mice [113]. In humans, physiological carnosine and β-alanine content is lower in muscle, red blood cells, and in urine of women [116–119]. Muscle carnosine levels show a greater increase in male subjects than in female subjects through puberty [119]. Upon carnosine supplementation, the increase in red blood cell carnosine content is less pronounced in women than in men, and carnosine excretion is higher in female subjects [118].

## Carnosine at Different Stages of Life

Significant changes in the metabolism of carnosine occur throughout human development, and CN1 seems to be a very important player in this process. In newborns, little to no CN1 is detectable in serum; CN1 levels then gradually increase from 1 year of age until adolescence [120]. At around 15 years of age, plasma/serum CN1 levels are comparable to those seen in adults [121, 122]. During aging, CN1 activity increases in serum and cerebrospinal fluid [16], while carnosine concentrations in muscles and blood decline [123, 124]. Interestingly, homocarnosine levels in cerebrospinal fluid of children are higher than in adults [125], with peak concentrations at around 5 years of age [126]. Age-related patterns in the levels and metabolism of carnosine and other histidinic dipeptides also occur in other species, including rodents [124], horses [127] and cattle [128]. Interestingly, PEPT2 may also be expressed differently during development and aging, according to a thorough review by Wang and collaborators [129].

The physiological roles of carnosine in earlier stages of development are not yet defined. Supplementation to young (up to 6-week-old) rodents does not seem to change body weight or food and water intake, as well as biochemical parameters [130, 131] or socialization [132]. However, supplementation with this dipeptide in young animals may result in deleterious effects for the muscles, as shown for rats (decreased activity of mitochondrial electron transport chain complexes [71]) and pigs (increased phosphorylation of mTOR [133]). In mice, supplementation of dams with carnosine during gestation leads to improvement of performance in motor skill tests in the litter. However, the pups also show lower proprioception scores, along with altered redox parameters in serum [134]. In non-mammalian species, exposure of zebrafish embryos to high carnosine concentrations leads to delayed development [135].

Animal models lacking carnosine synthetase present undetectable levels of histidine dipeptides. Despite unaltered olfactory bulb development and function until adulthood, carnosine synthetase knockout animals show reduced olfactory sensitivity upon aging [136]. These animals also present abnormal function in cardiac muscle [137], as well as milder defects in skeletal muscle development and function [136, 138, 139]. Moreover, carnosine synthetase knockout mice are more susceptible to neuroinflammation [140].

Alterations in carnosine metabolism have also been described in some pathological conditions at different stages of life. Lower carnosine synthetase expression has been reported in demyelinating lesions afflicting adult patients [141, 142] and in muscles of male and female oncologic patients with upper gastrointestinal cancer [5]. Carnosine is higher in blood samples from children with autism spectrum disorder in comparison with age-matched controls [143]. Increased CN1 content is found in cerebrospinal fluid of patients with Parkinson’s and Alzheimer’s diseases [144]. Carnosinase activity increases in patients undergoing pharmacological treatment for dementia, as well as in individuals with dementia who exercise regularly (but not in patients with dementia who are sedentary or not under treatment with dementia medication). Interestingly, CN1 activity in serum of patients with dementia correlates negatively with disease duration [145]. Mutations leading to decreased serum CN1 activity have been associated with protection against diabetic nephropathy [146], but do not impact longevity or coronary heart disease [147]. However, reduced CN1 is a hallmark of carnosinemia [148] (see below in Sect. 10) and has also been detected in other central nervous system diseases [149], as well as in patients with muscular dystrophy [120] and severe heart failure [150]. In Zucker diabetic fatty rats, carnosine levels are lower in heart but higher in plasma, compared to Sprague Dawley controls [151].

## Carnosine and the Brain: Lessons from Animal Studies

There is increasing interest in the roles of carnosine and related compounds in the brain. Brain carnosine metabolism can differ depending on the stage of development. Carnosine levels are progressively modulated at embryonic stages in the brain. In rat olfactory bulb, carnosine is absent at embryonic day (E) 15 but shows increasing scattered positivity in neurons at E17 and E20 [152]. Meanwhile, whole-brain carnosine content decreases slightly between E15.5 and E19.5 in mice [153]. In the developing post-natal rat brain, De Marchis and colleagues showed that carnosine-like immunoreactivity distribution begins in the posterior region and then spreads anteriorly. At early stages (postnatal day 6 – P6), carnosine is predominantly present in the brain stem, spreading towards tegmentum at P9, towards cerebellum, hypothalamus and corpus callosum at P12, and towards cerebral cortex and olfactory bulb at P21 [154]. On the other hand, earlier evidence suggests that carnosine may already be present in olfactory bulb at embryonic and neonatal stages [155, 156]. Interestingly, in adult murine brain, homocarnosine is the most abundant histidine dipeptide in most structures, except for the olfactory bulb, where carnosine predominates [3].

**Fig. 1:**
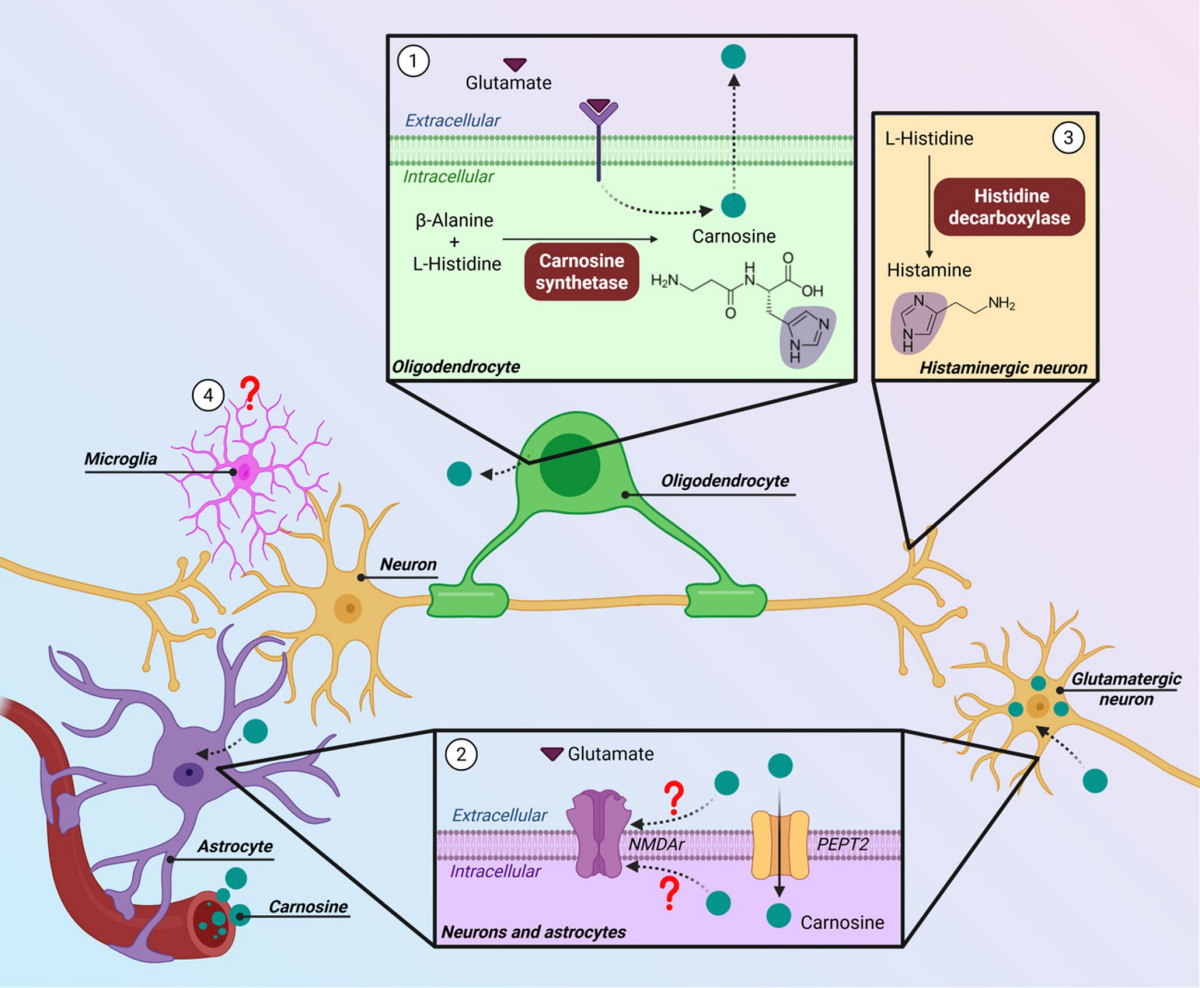
Carnosine metabolism in mammalian brain. (1) Oligodendrocytes (green cell) express carnosine synthetase, which catalyzes carnosine synthesis from β-alanine and L-histidine in these cells. The release of carnosine (green circles) from oligodendrocytes may be influenced by glutamate signaling. (2) Extracellular carnosine is taken up by astrocytes (purple cell) and neurons (yellow cells) mainly via peptide transporter 2 (PEPT2). Carnosine may exert some effects through NMDA receptors. (3) Histaminergic system may also be impacted by carnosine, given its structural similarity with histamine (highlighted imidazolic ring). (4) To date, carnosine metabolism and expression of PEPT2 in microglia (pink cell) are still unknown. Created with BioRender. Ferreira, G. (2026) https://BioRender.com/f7g7xve

Metabolic compartmentation has been proposed for carnosine in the brain (Fig. 1). In the olfactory system, carnosine is restricted to mature neurons. Non-olfactory carnosine was initially described as being confined to glial cells, primarily in astrocytes and Bergman glia [155]. However, when astrocytes and oligodendrocytes are cultured together, carnosine synthesis seems to occur in oligodendrocytes, and it is taken up by astrocytes [157]. A recent study showed that neurons can also take up carnosine [28]. Interestingly, the *Km* of PEPT2 for carnosine in primary neuronal cultures (119 µM) is higher than in primary astrocytic cultures (43 µM*)*, so neuronal carnosine uptake is favored at higher concentrations [27, 28].

Identifying the actions of carnosine in the brain is essential for understanding its relevance in neurophysiology and its therapeutic potential. Carnosine co-localizes with glutamate in neurons of mice [158] and amphibians [159], and the release of both carnosine and β-alanine from primary cultures of rat oligodendrocytes can be mediated by glutamate receptors [160]. Interestingly, morphologic and redox alterations induced by carnosine in cultured astrocytes from rats can be at least partially prevented by the non-competitive antagonist of NMDA receptors MK-801, thus suggesting an involvement of NMDA receptors [46]. Carnosine regulates the binding of serotonin to its receptor in rat brain [161] and modulates the activity of monoamine oxidase-A in different rat brain regions [162]. Given its Zn^2^-chelating properties, carnosine can also impact glycine-induced currents through glycine receptors in cultured hippocampal neurons from mouse embryos [163]. In addition, glutamic acid decarboxylase and GABA transporter (GABA-T) can be inhibited by both carnosine and homocarnosine in rat brain synaptosomes [164]. Thus, carnosine may interact with several neurotransmitter systems.

As shown in a report based on carnosine synthetase knockout mice, exogenous carnosine (given orally) reaches the brain [4]. In line with this, Macedo and collaborators have shown that brain bioenergetics can be impacted by intraperitoneally administered carnosine [72]. Despite the high plasma CN1 activity in humans, a recent study indicated that oral supplementation with high doses of carnosine to humans can result in a transiently higher brain carnosine content [98]. Improved high-level cognitive performance is also suggested for healthy individuals receiving carnosine supplementation [165]. Taken together, these data suggest that peripheral carnosine may reach the brain directly and influence brain function.

## Carnosinemia

Carnosinemia (Online Mendelian Inheritance in Man - OMIM #212200) is an autosomal recessive inborn error of metabolism caused by mutations in the *CNDP1* gene, leading to deficient CN1 activity [148, 166]. It is characterized by high carnosine levels in tissues and body fluids such as plasma, cerebrospinal fluid, and urine [148, 167–169]. Affected patients present with tremors, hypotonia, seizures, and severe psychomotor and cognitive delay. Signs and symptoms may appear in early childhood (as early as 3 months of age) and develop with age [168–170]. Diagnosis consists of assessing amino-acid levels in plasma or urine, and measuring CN1 activity [171, 172]. Treatment focuses on a carnosine-restricted diet and on controlling the symptoms (especially seizures) [168]. There is presently no cure for this disorder. This is a rare disease, and its current prevalence is unknown. It is believed that the high carnosine concentrations contribute to the triggering, perpetuation, or aggravation of the signs and symptoms. Nonetheless, the precise pathomechanisms of carnosinemia have not yet been elucidated. A recent study showed that carnosine was the most abundant dipeptide in plasma (and the third most abundant dipeptide in cerebrospinal fluid) of children with neurological or metabolic conditions of unknown origin, including 23 children with epilepsy [173]. In addition, possible impacts of other imidazole dipeptides in this disease cannot be ruled out. There is evidence that anserine is significantly higher in cerebrospinal fluid of children with epilepsy than of those without epilepsy [173], and homocarnosine has also been associated with epilepsy in children [174].

## Future Directions

**Fig. 2:**
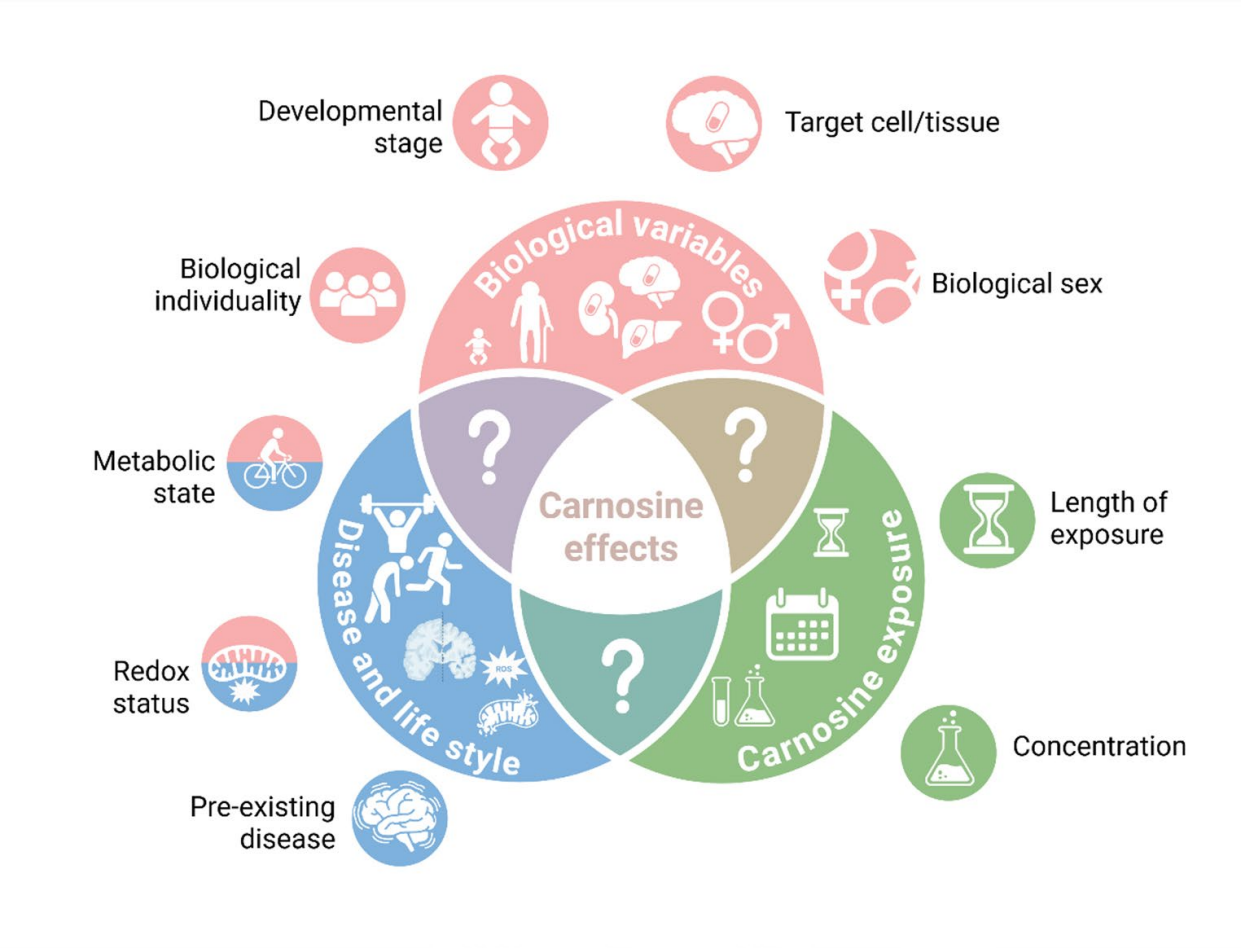
Potential factors influencing carnosine effects. Multiple physiological and pathological variables may interact and modulate carnosine effects, including stage of development, target cell/tissue, biological sex, metabolic and redox state, biological individuality, and pre-existing conditions/diseases. The length of exposure and the concentration/dosage of carnosine may also contribute to the plurality of carnosine effects. Created with BioRender. Ferreira, G. (2026) https://BioRender.com/f7g7xve

It is undisputed that carnosine is an interesting molecule, and in some respects a promising one. However, there are still gaps in the understanding of its actions in a non-pathological context and in how those actions might vary in different contexts (stage of development, tissue, conditions, sexes, metabolic state, etc.) (Fig. 2). These may have important implications for its use as a supplement or as a therapeutic agent. Data discussed in this study support that carnosine supplementation should be carefully monitored in earlier stages of development, as its developmentally variable physiology may be related to different responses, in comparison to adult or elderly individuals (especially in the brain). These responses may also be linked to the neurological dysfunction of carnosinemia. Thus, interrogating the potential of carnosine could contribute to its better use, as well as increasing our understanding of carnosine physiology and its involvement in diseases.

## Data Availability

No data was produced for the research described in the article.
